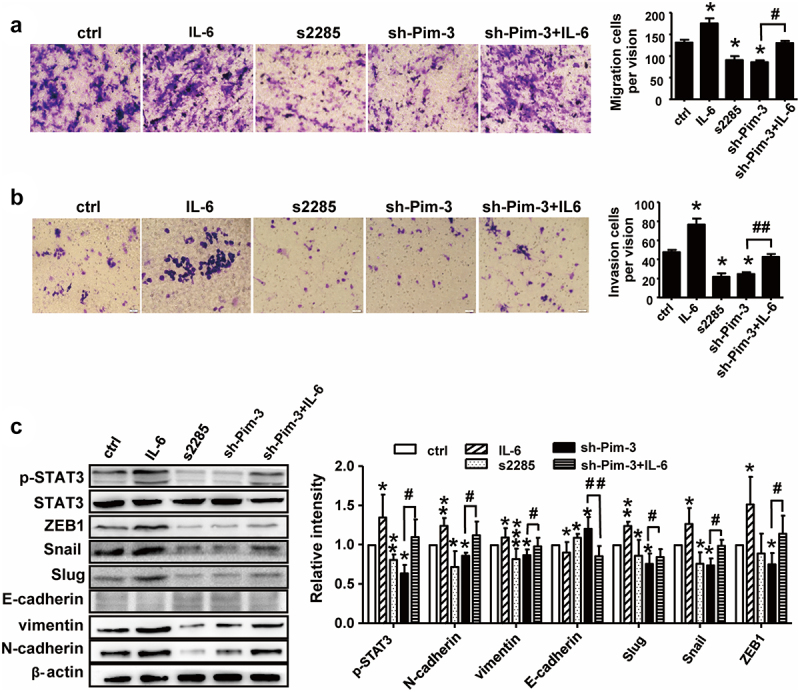# Correction

**DOI:** 10.1080/15384047.2025.2500190

**Published:** 2025-05-14

**Article title**: Pim-3 enhances melanoma cell migration and invasion by promoting STAT3 phosphorylation

**Authors**: Liu, J., Qu, X., Shao, L., Hu, Y., Lan, P., Guo, Q., Han, Q., Zhang, J., & Zhang C.

**Journal**: *Cancer Biology & Therapy*

**Bibliometrics**: Volume 19, Number 03, pages 160-168

**DOI**: https://doi.org/10.1080/15384047.2017.1414756

The authors regret that incorrect images appeared in Figures 3, 5, and 6 due to errors during the data arrangement process. Specifically, the representative cell transwell images were displayed incorrectly. The corrected versions of the figures are now provided below. This correction does not affect the original conclusions or any part of the text or figure legends. The authors apologize for any inconvenience caused.

**Figure 3:**
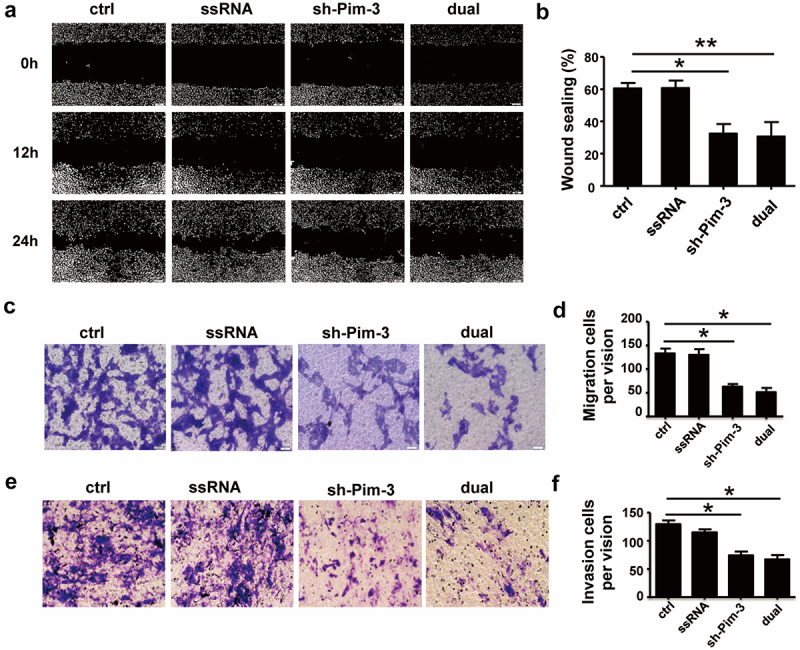

**Figure 5:**
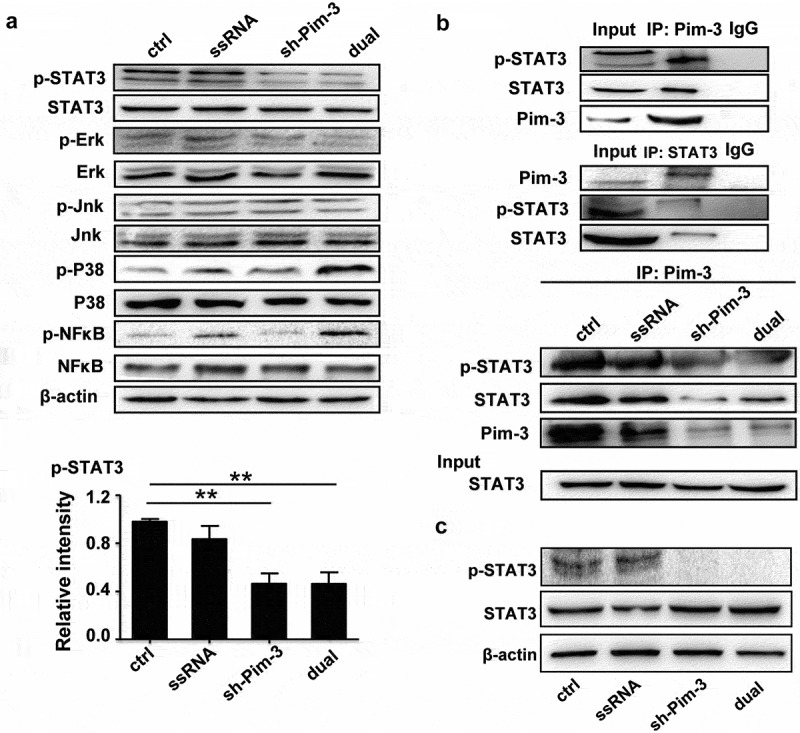

**Figure 6:**